# Factors affecting spirometry reference range in growing children

**DOI:** 10.12669/pjms.35.6.1212

**Published:** 2019

**Authors:** Sara Sadiq, Nadeem Ahmed Rizvi, Fahad Khalid Soleja, Muaz Abbasi

**Affiliations:** 1 Sara Sadiq, MBBS, M.Phil. Department of Physiology, CMH Institute of Medical Sciences, Bahawalpur, Pakistan; 2 Prof. Nadeem Ahmed Rizvi, MBBS, MCPS, MRCP, FRCP. Head of Chest Medicine, Jinnah Postgraduate Medical Centre Karachi, Pakistan; 3 Fahad Khalid Soleja, Undergraduate MBBS Student, Ziauddin University, Karachi, Pakistan; 4 Muaz Abbasi, Undergraduate MBBS Student, Ziauddin University, Karachi, Pakistan

**Keywords:** Pulmonary function test, Spirometry, Forced vital capacity, Regression analysis

## Abstract

**Objectives:**

To find out the association of weight, height and age with spirometry variables and to generate a regression equation by taking weight as an independent variable beside age and height among children and adolescents of Karachi.

**Methods:**

A modified form of ISSAC questionnaire was used. The spirometry variables recorded were Forced vital capacity (FVC), Forced expiratory volume in 1 second (FEV_1_), FEV_1_/FVC, Peak expiratory flow rate (PEF), Forced expiratory flow between 25% and 75% expired volume (FEF_25-75_). A person’s correlation coefficient among boys and girls were calculated for all spirometry variable considering age, height and weight as independent variables. The linear regression models were calculated.

**Results:**

The results reported a linear correlation of lung function variables with all three independent variables (i.e. p-value = 0.000), in which age and height manifested a strong positive correlation while weight reported a moderately significant correlation. All spirometry variables such as FVC, FEV_1_, PEF and FEF_25-75_ reported a significant coefficient of dependency and coefficient of correlation individually with age, height and weight.

**Conclusion:**

It is concluded that beside age, height and weight both also have significant correlation with lung volumes so these should be taken into account when using spirometry as a diagnostic test.

## INTRODUCTION

Globally respiratory tract diseases are considered as common cause for both morbidity and mortality.^1^ According to European Lung White Book the reason behind the frequent visit of children to hospital are these respiratory tract diseases which are accounting for about 25%.^2^ As Pakistan is a developing country with high prevalence of respiratory tract diseases. for accurate diagnosis of these respiratory diseases multiple lung function tests are used, among them spirometry is the gold standard one.^3^

Spirometry results give a clue about the level of morbidity and life expectancy. These results help the physician in making a decision regarding the nature of disease, its severity and probable response to medication.^4^-^6^ Spirometry just give an overview of general respiratory health in the same way as the recording of blood pressure reveals about the overall cardiovascular health.^7^ Interpretation of spirometry result is a key step for accurate diagnosis, which depends upon the specific reference range for that particular area and population. As the literature reported the reference range variations among population of different regions and the most probable reasons behind these variations might be those factors that have influences over the lung functions like age, sex, height, weight, ethnicity, socioeconomic status, cultural beliefs and biomass smoke exposure.^4^,^5^,^8^

Considering specifically south Asia, very few studies have been done to establish a normative spirometry range among children and adolescents by taking age and height as an independent variable.^9^,^10^ Among developing countries specially in Pakistan, insignificant number of studies have been done for adult population but none of them reported any of the normative spirometry values for the children and adolescents.^6^,^11^,^12^ Because of this physician are diagnosing the children and adolescents on the basis of Polgar reference values, in which height is an independent variable.^13^ On the other hand, few of the studies considered weight also as an independent variable beside height and age, while developing regression equation.^10^,^14^ This created a confusion that which of the factor should keep as an independent variable while generating reference range. The association of age and height with the spirometry variables are established up to some extent but the exact correlation of weight is not yet cleared among Asian children and adolescents.^15^ So the aims of current study are to find out the association of weight, height and age with the spirometry variables and to generate a regression equation by taking weight as an independent variable beside age and height among children and adolescents of Karachi.

## METHODS

A cross-sectional study started from the month of April 2017 up to October 2017. Data collection was done from the different primary, middle, secondary, higher secondary schools and Maddarssa of Karachi. Study got approval (Ref.No. 0170617SSMP) from the Ethical Review Committee of Ziauddin University and Hospital. Informed written consent was taken from both, the school authorities and parents while assent from children and adolescents. Sampling technique used was multistage technique in which during first stage eight schools and a Maddarssa was randomly selected from all districts of Karachi, considering socioeconomic strata. During the second stage of sampling technique, the children and adolescents of required age group were randomly selected from those schools.

Exclusion criteria followed for the study was (1) children younger than 7 years (2) any trauma that affected respiratory system (3) diagnosed cases of asthma, wheezing, allergic rhinitis, or any significant respiratory tract disease (4) diagnosed cases of congenital heart diseases (5) diagnosed cases of muscular disorders including Duchene muscular dystrophy (6) individual with bronchodilator therapy (7) any deformity of chest wall (8) active smokers. A modified form of International Study of Asthma and Allergies in Childhood (ISAAC) Questionnaire was used. Height and weight was noted. Detailed general physical and systemic examination especially respiratory system examination was done to exclude the children and adolescents with any disease that can deteriorate the spirometry reference range.

For taking spirometry variables, the instrument used was Vitalograph-alpha. It was calibrated before performing the procedure. A trained doctor supervised the procedure by following American thoracic society/European respiratory society (ATS/ERS) task force 2005 standardization guidelines. The procedure was performed in sitting position; nose was pinched by using a nose clip. Minimum of three and maximum of eight maneuvers were performed. Spirometry graph was observed, considering acceptability, repeatability and reproducibility criteria’s of ATS/ERS task force 2005 standardization guidelines. The spirometry variables recorded were Forced vital capacity (FVC), Forced expiratory volume in 1 second (FEV_1_), FEV_1_/FVC, Peak expiratory flow rate (PEF), Forced expiratory flow between 25% and 75% expired volume (FEF_25-75_).

Data was analyzed by using 20^th^ version of Statistical program for social science (SPSS). All the quantitative variables were mentioned as mean with standard deviation. A person’s correlation coefficient among boys and girls were calculated for all spirometry variable including FVC, FEV_1_, PEF and FEF_25-75_, considering age, height and weight as independent variables. A scatter plot with regression line was drawn to find out the association. The linear regression models were calculated for all pulmonary variables with the age, height and weight. Data with p<0.05 were considered as statistically significant.

## RESULTS

About 1085 participants were enrolled in the study but some of the participants were excluded because of either active smoking or performed unsatisfactory test or couldn’t follow the acceptability guidelines of ATS/ERS task force 2005. So after excluding, finally 751 participants were analyzed. The main demographic variables like age, height, weight and spirometry variables including FVC, FEV_1_, PEF and FEF_25-75_ are presented in Table-I in the form of mean and standard deviation. The table also reported variation among the mean values of demographic variables of boys and girls.

**Table I:** Mean and Standard deviation of Demographic and Pulmonary function variables.

	Mean ± SD (n = 751)	Boys (n = 484)	Girls (n = 267)
Age	12.96 ± 2.8	13.1 ± 2.7	12.66 ± 2.8
Height (cm)	150.2 ± 15.8	152.3 ± 16.7	146.4 ± 13.3
Weight (Kg)	44.2 ± 16.6	45.3 ± 17.3	42.2 ± 15
BMI	19 ± 4.4	18.9 ± 4.3	19.2 ± 4.5
FVC	2.21 ± 0.75	2.28 ± 0.753	2.10 ± 0.74
FEV1	2.08 ± 0.73	2.13 ± 0.726	1.97 ± 0.73
FEV1/FVC	92.9 ± 4.7	92.93 ± 4.78	92.89 ± 4.49
PEF	231.3 ± 70.5	236.6 ± 73.59	221.6 ± 63.6
FEF_25-75_	2.68 ± 1.2	2.78 ± 1.26	2.52 ± 1.06

**Fig. 1:**
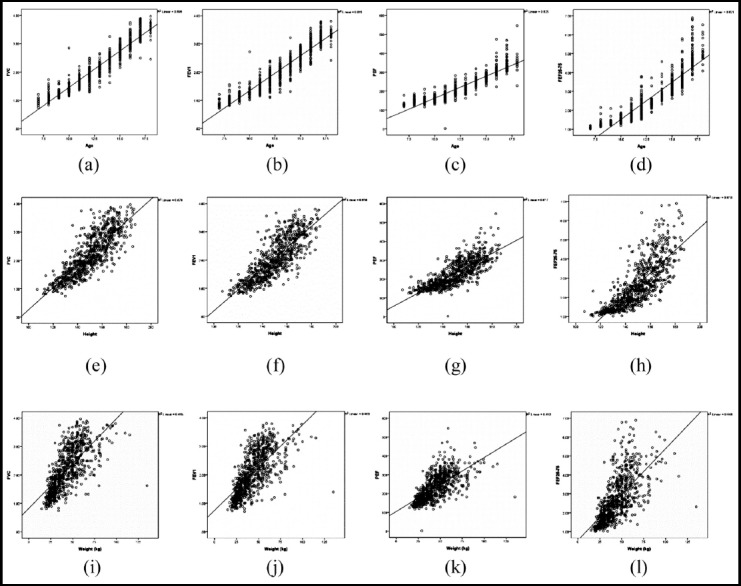
Correlation of (1)age with (a)FVC (b)FEV_1_ (c)PEF (d)FEF_25-75_ (2)height with (e)FVC (f)FEV_1_ (g)PEF (g)FEF_25-75_ (3)weight with (i)FVC (j)FEV_1_ (k)PEF (l)FEF_25-75_

A person’s correlation coefficient among boys and girls were calculated for all spirometry variable including FVC, FEV_1_, PEF and FEF_25-75_, considering age, height and weight as independent variables, as shown in Table-II. The results reported a linear correlation of lung function variables with all three independent variables (i.e. p-value = 0.000), in which age and height manifested a strong positive correlation while weight reported a moderately significant correlation. The regression line in scatter plot of spirometry variables with independent variable like age, height and weight displayed a linear association as shown in Fig. 1 respectively. The graphs are evident of increase in lung function with increasing age, height and weight in children and adolescents.

**Table II:** Correlation coefficients of age, height and weight with spirometry variables among boys and girls.

	Boys Correlation coefficient with			Girls Correlation coefficient with		
	Age	Height	Weight	Age	Height	Weight

FVC	0.944	0.840	0.713	0.955	0.787	0.655
FEV_1_	0.937	0.831	0.702	0.941	0.774	0.633
PEF	0.892	0.790	0.663	0.949	0.771	0.687
FEF_25-75_	0.898	0.794	0.670	0.936	0.742	0.659

All those factors were taking into account that have an influence over the spirometry normative values like age, sex, height weight, socioeconomic status cultural factors and biomass smoke exposure, the results reported that the three main factors including age, height and weight should be considered as independent factors. So by putting age, height and weight as independent variables, the regression equation were calculated (p-value = 0.000) as mentioned in Table-III. This regression equation can be considered as a best predictive model for calculating pulmonary function among children and adolescents of Pakistan as it reported a significant coefficient of dependency and coefficient of correlation individually with age, height and weight.

**Table III:** Regression model of Spirometry variables with Age, Height and Weight.

	R	R^2^	Regression equation	p-value
FVC	0.949	0.901	(-1.431)+0.234(A)+0.004(H)+0.001(W)	0.000
FEV_1_	0.940	0.883	(-1.444)+0.230(A)+0.003(H)+0.001(W)	0.000
PEF	0.910	0.828	(-87.804)+21.164(A)+0.238(H)+0.208(W)	0.000
FEF_25-75_	0.908	0.824	(-2.670)+0.363(A)+0.003(H)+0.004(W)	0.000

## DISCUSSION

Spirometry is one of the gold standard test for diagnosing respiratory diseases so an authentic region specific reference range is a crucial need.^16^ As there are multiple causal factors including age, sex, height weight, socioeconomic status cultural factors and biomass smoke exposure, that are responsible for significant variations in the range of spirometry variables among the populations. This created a confusion that which of the factor should keep as an independent variable while generating predictive equation^15^ and the current study developed a regression equation for children and adolescents, by putting age, height and weight as independent variables.

Considering specifically age as an independent variable, multiple studies reported a linear type of correlation among children and adolescents^14^,^17^ and the current study manifest a strong positive correlation of all spirometry variables including FVC, FEV_1_, PEF and FEF_25-75_ with age. It is stated that the lung volumes increase progressively along with age due to increase muscularity as well as increase in the size of chest cavity, resulting in increased lung compliance.^17^ But this is only true for children and adolescents, but not for the adults, who shows negative correlation of lung function with age because of the decrease elastic recoil of lung and smaller airways.^18^

Looking over the height as an independent variable, it has a linear type of correlation with the spirometry variables^19^-^21^ and the current study also shows a highly significant correlation of height with all pulmonary variables. Ma Y-N et al reported an increase in pulmonary volumes and capacities with increasing height and concluded a direct association of lung function variables with height.^22^ Because of this strong correlation of height, a Chinese study kept height only as a main independent variable, leaving behind age and weight.^10^

Literature review revealed a wide variation among the results when taking weight while establishing spirometry reference range. As some of the studies reported non-significant association of weight with the spirometry variables^21^,^23^ while other had strongly significant correlation of weight and spirometry variables.^17^,^20^,^24^ The current study favored the finding by showing a moderately significant correlation of weight with spirometry variables.

## CONCLUSION

It is concluded that beside age, height and weight both having significant correlation with lung volumes so these should be taken into account when using spirometry as a diagnostic test. The regression equation of current study can be considered as a best predictive model for calculating pulmonary function among children and adolescents of Pakistan.

### Authors’ Contribution:

**SS:** Conceived, designed and did statistical analysis & editing of manuscript.

**SS, FKS and MA:** Did data collection and manuscript writing.

**NAR:** Did review and final approval of manuscript.

## References

[1] Stanojevic S, Wade A, Stocks J (2010). Reference values for lung function:past, present and future. Eur Respir J.

[2] Gibson GJ, Loddenkemper R, Lundback B, Sibille Y (2013). Respiratory health and disease in Europe:the new European Lung White Book. Eur Respiratory Soc 2013. Eur Respir J.

[3] Lum S, Bountziouka V, Quanjer P, Sonnappa S, Wade A, Beardsmore C (2016). Challenges in collating spirometry reference data for South-Asian children:an observational study. PloS One.

[4] Strippoli M-PF, Kuehni CE, Dogaru CM, Spycher BD, McNally T, Silverman M (2013). Etiology of ethnic differences in childhood spirometry. Pediatrics.

[5] Quanjer PH, Tammeling G, Cotes J, Pedersen O, Peslin R, Yernault J (1993). Lung volumes and forced ventilatory flows. Eur Respiratory Soc. Eur Respir J.

[6] Nadeem M, Raza S, Malik M (1999). Ventilatory function of healthy, urban, non smoking, Pakistani young adults aged 18–24 years. Respir Med.

[7] (2012). American Thoracic Society Standardization of Spirometry, 1994 Update.

[8] Mohammed J, Maiwada Sa, Sumaila FG (2015). Relationship between anthropometric variables and lung function parameters among primary school children. Ann Niger Med.

[9] Kumar R, Seibold MA, Aldrich MC, Williams LK, Reiner AP, Colangelo L (2010). Genetic ancestry in lung-function predictions. N Engl J Med.

[10] Jiang M, Gao Y, Zhong NS, Chen WQ, Guan WJ, Zheng JP (2015). Spirometric reference values for healthy Han children aged 5–15 years in Guangzhou, southern China. Pediatr Pulmonol.

[11] Memon MA, Sandila MP, Ahmed ST (2007). Spirometric reference values in healthy, non-smoking, urban Pakistani population. J Pak Med Assoc.

[12] Bhatti U, Rani K, Memon MQ (2014). Variation in lung volumes and capacities among young males in relation to height. J Ayub Med Coll Abbottabad.

[13] Quanjer PH, Borsboom G, Brunekreef B, Zach M, Forche G, Cotes J (1995). Spirometric reference values for white European children and adolescents:Polgar revisited. Pediatr Pulmonol.

[14] Budhiraja S, Singh D, Pooni PA, Dhooria GS (2010). Pulmonary functions in normal school children in the age group of 6–15 years in north India. Iran J Pediatr.

[15] Sadiq S, Ahmed ST, Fawad B (2018). Collating Spirometry reference values in Asian children and Adolescents;puzzle out the reasons for variations. Pak J Med Sci.

[16] Pellegrino R, Viegi G, Brusasco V, Crapo R, Burgos F, Casaburi R (2005). Interpretative strategies for lung function tests. Eur Respir J.

[17] Doctor TH, Trivedi SS, Chudasama RK (2010). Pulmonary function test in healthy school children of 8 to 14 years age in south Gujarat region, India. Lung India: official organ of Indian Chest Soc.

[18] Baarends E, Schols A, Mostert R, Wouters E (1997). Peak exercise response in relation to tissue depletion in patients with chronic obstructive pulmonary disease. Eur Respir J.

[19] Takase M, Sakata H, Shikada M, Tatara K, Fukushima T, Miyakawa T (2013). Development of reference equations for spirometry in Japanese children aged 6–18 years. Pediatr Pulmonol.

[20] Singh V, Kurrey V, Khandwal O, Phulijhele S (2014). Evaluation of Lung Function by Spirometry in 12-14 yrs Adolescents in schools of Raipur city Chhattisgarh. Inter J Med Sci Res Prac.

[21] Chhabra SK, Kumar R, Mittal V (2016). Prediction equations for spirometry for children from northern India. Indian Pediatr.

[22] Ma Y-N, Wang J, Dong G-H, Liu M-M, Wang D, Liu Y-Q (2013). Predictive equations using regression analysis of pulmonary function for healthy children in Northeast China. PloS one.

[23] Al-Riyami BM, Al-Rawas OA, Hassan MO (2004). Normal spirometric reference values for Omani children and adolescents. Respirology.

[24] Chhabra S, Vijayan V, Rahman M, Mittal V, Singh P (2012). Regression equations for spirometry in children aged 6 to 17 years in Delhi region. Ind J Chest Dis Allied Sci.

